# Training medical students to manage difficult circumstances- a curriculum for resilience and resourcefulness?

**DOI:** 10.1186/s12909-019-1712-x

**Published:** 2019-07-25

**Authors:** Barry Wright, Joseph Richmond Mynett

**Affiliations:** Hull York Medical School, University of York, John Hughlings Jackson Building, Heslington, York, YO10 5DD UK

**Keywords:** Resilience, Coping, Burnout, Undergraduate, Medicine, Curriculum, Training, Evaluation, Education, Medical school, Medical students

## Abstract

**Background:**

In response to the growing prevalence of physical and emotional burnout amongst medical students and practicing physicians, we sought to find a new methodology to scope a five-year undergraduate curriculum in detail to assess for teaching, learning objectives and experiences that seek to promote resilience in medical students. This was undertaken to test whether this methodology would enable curriculum discussions to enhance training for future cohorts through the introduction of a curriculum dedicated to the development of resilience and resourcefulness.

**Methods:**

Based on literature review, a rating-scale was devised to generate quantitative data in four key areas of resilience; internal resources, lifestyle factors, external resources (self-mediated) and external resources (agent mediated). This scale was used to evaluate the entire five-year undergraduate curriculum of a medical school in the north of England through systematic evaluation of learning outcomes and planned activities. The methodology used was a four-stage process including i) identifying the learning objectives, ii) mapping them onto the criteria outlined, iii) assessing them against clear objective standards (planned, explicit, universal and quantifiable), and iv) rating data collected.

**Results:**

The evaluation provided a clear, quantitative overview of the curriculum in terms of resilience building. Strengths and gaps were identified and work was undertaken leading to suggestions for change. This facilitated helpful discussions with course leaders and planners, received universally positive feedback and led to new learning objectives, activities and experiences that have been identified and begun to be implemented.

**Conclusions:**

**“**The HYMS CARE Criteria” and our methodology for assessing it in a medical school curriculum context, offers a valuable perspective to aid the planning of improvements in curricula. This model for scoping and structuring resilience related learning experiences is offered for consideration by other schools.

## Background

### Physician and medical student burnout

Burnout describes a reaction to ongoing stress, a state of emotional exhaustion that can lead to reduced perceived or actual personal accomplishment [1, 2]. A meta-analysis of medical students in the United States suggests that both physicians in training and practicing physicians experience high rates of burnout, whilst factors contributing to burnout such as depersonalisation and low personal accomplishment were found to be highly prevalent in a similar UK study [3, 4].

Following a scoping literature review to explore the effects of burnout (including number of sick leave days, work ability, and intent to either keep practicing or change jobs) the majority of studies we identified indicated a negative relationship between burnout and safe and productive practice [5]. One factor that could reduce levels of burnout is resilience. Higher resilience levels are associated with lower levels of burnout and better tolerance of uncertainty [6]. Conversely, a wide range of issues are associated with low resilience levels, including stress, depression and substance misuse, all of which can also have a negative impact on patient care [7].

### Defining resilience

The term ‘resilience’ has been interpreted in many different ways. In order to focus our curriculum evaluation methodology, we have framed resilience as the mechanisms by which an individual might be equipped to engage with stressors with minimum negative impact, whilst experiencing personal growth and leading to the development of new coping mechanisms [8].

This context allows literature-based identification and exploration of the factors that contribute to building this form of coping resilience, and subsequently the development of a means to identify these within a curriculum.

### What factors affect resilience in medical school?

#### Internal resources

The current research base looking at resilience in medical school appears to follow several distinct themes relating to coping and wellbeing. The most prominent of these is the building of internal resources. A regression analysis in a study of Chinese medical students found that a resilience scale moderated negative life events and mental health problems where the scale essentially measures ability to endure difficult circumstances [9, 10]. The teaching of communication skills in difficult circumstances (e.g. breaking bad news) is widely seen as positive and valued by medical students in equipping them with skills for improved coping and reduced stress in those situations, supporting the idea that practical solutions can be an effective means to enhance resourcefulness and coping in difficult situations [11]. Using an applied literature search, Dunn and colleagues proposed a coping reservoir model that can be replenished or drained [12]. This work focused on personal traits, temperament and coping style, all of which can be seen as internal resources [12].

#### Lifestyle

Another theme that has been explored is the effect of lifestyle factors. Healthy spare time experiences have been shown to promote resilience in young people at school, suggesting that this may also be the case in medical school with good personal life and work life balance leading to improved satisfaction at graduation [13, 14].

#### Self-efficacy and seeking to employ external resources

Howe and colleagues suggested that important elements of resilience in medical training included self-efficacy, ability to engage support, self-control, learning from difficulties and tenacity in the face of challenges [15]. Research also suggests that good social support and developing active coping strategies play a protective role, encompassing not just lifestyle factors but also connectedness and supportive social frameworks [9, 16, 17]. A longitudinal observational study at one medical school investigated the effect of maintaining physical activity on resilience, with the results suggesting that promotion and provision of physical activity may encourage improved general health and therefore resilience [18].

#### Agent mediated resources and training

There is an emerging body of evidence that training to improve resilience may be helpful; however, the methodologies of these studies is limited [19, 20]. More comprehensively, a broad scale, holistic approach applied at the Saint Louis University School of Medicine based on adjustments to course content such as timetabling, grading and electives, combined with specific resilience and mindfulness content was shown to reduce depression, anxiety and stress symptoms in participating students [21]. This supports the idea that bespoke resilience teaching incorporating a comprehensive range of factors could produce positive results with the correct execution. There is also evidence that suggests tailored training of individual skills such as empathy and communication can be highly effective, however these are shown in isolation and not as part of a wider, holistic training programme [22, 23].

Based upon this evidence, there are a number of aspects and perspectives to consider when beginning to understand or construct a curriculum that wishes to address the issue of resilience. This implies the endeavour should be ambitious in scope, fully integrated into the curriculum and become part of the journey of life-long learning. Stand-alone training (e.g. in mindfulness) only constitutes a small part of a bigger picture; however, a more considered and comprehensive approach should arm students with the tools to cope more effectively. An ability to assess a curriculum by taking a range of learning experiences into account therefore becomes important.

## Methods

### Aim

In the spirit of searching out better ways of training the doctors of the future from a supportive framework, we sought to devise an evaluation tool that could be used to fulfil the following objectives:

1. Identify a comprehensive list of factors that contribute to resilience building
2. Evaluate an existing medical school curriculum for teaching and experiences that promote or teach resilience
3. Identify areas of strength and opportunities for improvement within a curriculum
4. Provide data that can be used as a basis for future planning and discussion within the medical school
5. Extract all elements of resilience building from a medical school curriculum and assemble and articulate a standalone curriculum across time to enhance resilience and resourcefulness

### Assessment criteria concept

In order to create a comprehensive resilience assessment tool, it was first necessary to seek out the factors shown to contribute to resilience within the relevant general literature, and the specific literatures relating to factors affecting medical students as discussed in the previous background section. Following this we put together an expert group of educators, clinicians and students, including expertise in psychiatry, psychology, palliative care, general practice, public health, student support services and academia. This group discussed and agreed upon the principles on which the curriculum would be assessed based on this literature. Following the assessment, discussed recommendations to create new learning objectives and experiences to enhance the curriculum based upon the data.

### Criteria design

The organisation of our assessment tool, the HYMS CARE criteria (HCC) (Table 1), was based upon grouping of the resilience factors discussed in the literature, allowing us to design a catalogue of itemised factors in the context of larger themes. In order to generate versatile data sets, we implemented three levels of organisation; 31 individual elements, 10 groups of elements and 4 overall themes, all of which can be visualised independently following a curriculum evaluation.

**Table 1:** The HYMS CARE criteria

1–4: Internal Resources			
1	Developing empathy skills	1.1	Personality/temperament/optimism/openness
		1.2	Empathy
2	Developing insight	2.1	Reflectiveness
		2.2	Self-awareness/insightfulness
3	Developing resourcefulness	3.1	Problem solving/Social problem solving
		3.2	Exercising judgment/weighing up/responsibility mapping/prioritisation
		3.3	Exec function/organisational abilities
		3.4	Developing ethical compass
		3.5	Confidence/autonomy
4	Team work and communication	4.1	Team working ability
		4.2	Communication
5–7: Lifestyle factors			
5	Physical health self-efficacy	5.1	Nutrition/sleep/physical activity
		5.2	Health behaviours
		5.3	Personal safety
6	Mental health self-efficacy	6.1	Self-esteem/self-compassion
		6.2	Managing emotions
		6.3	Taught skills; mindfulness/relaxation
7	Achieving work-life balance	7.1	Hobbies
		7.2	Routine/ stability
8–9: External resources (self-mediated)			
8	Building support networks	8.1	Proactivity
		8.2	Enlisting academic help
		8.3	Enlisting pastoral support
		8.4	Connectedness and belonging/giving and receiving care
9	Learning effective use of external resources	9.1	Career/CPD planning
		9.2	Time management & prioritisation
		9.3	Searching skills/literacy
		9.4	Planning abilities
10: External resources (agent-mediated)			
10	Provision of external resources	10.1	Mentoring/Student support
		10.2	Information/Academic support
		10.3	Supportive systems & processes
		10.4	Fostering connectedness

The themes chosen were designed to isolate the differing forces acting upon resilience and resourcefulness, from an individual’s impact on their environment to the environmental impact on the individual. This resulted in four distinct categories of resilience building; internal resources, lifestyle factors, external resources (self-mediated) and external resources (Agent mediated). “Internal resources” represents the personal traits and skills of the individual. These include factors such as empathy, personality and temperament, and ethical development [24, 25]. “Lifestyle factors” are the elements used to strengthen work-life balance and promote self-care, encompassing elements such as self-compassion, positive self-beliefs, maintaining physical health and maintaining energy levels [26–29]. “External resources (self-mediated)” refers to the ability to identify and interact with support networks and institutional frameworks. This includes factors such as connectedness and actively seeking out and enlisting support [30–32]. The final category, “External factors (agent mediated)” refers to the influence of the institution on the individual through provision of resources and support. This category is distinct in that it reflects the structure of the organisation, in this case a medical school, as opposed to identifying skills that can be enhanced within the individual students.

The HCC is intended as an itemised inventory of factors believed to influence resilience and resourcefulness among practicing medics and medical students. It is not intended as a definitive exposition of resilience, rather a tool that can be used to navigate areas of interest and assess current curricula to enable meaningful discussion about strengths and opportunities for improvement.

The CARE criteria were coined in the expert workshop groups with the acronym representing Compassion to self and others, Adaptability, Resourcefulness and Emotional wellbeing. They are listed in Table 1.

In order to evaluate this tool, we set out to apply a robust methodology in the assessment of a medical school curriculum using a four-stage process including i) identifying the learning objectives, ii) mapping them onto the criteria outlined, iii) assessing them against clear objective standards (planned, explicit, universal and quantifiable), and iv) rating data collected.

## Methodology for identifying resilience building in a medical school curriculum using the CARE criteria

### The curriculum assessed

A 5-year undergraduate MBBS programme in a medical school in the North of England, UK.

### Identifying relevant course content

The medical school used in this study arranges teaching into three sequential Phases: Phase I (years 1&2) builds a knowledge base predominantly through classroom teaching, Phase II (years 3&4) places students in clinical environments full time, focussing on clinician teaching, self-directed study and topic-based masterclasses, and Phase III (Year 5) enlists students as junior members of multidisciplinary teams, rotating through different specialities. The curriculum was assessed in three stages, correlating to these course phases. In collaboration with senior course leaders and administrative staff, relevant documents outlining course content for each phase were identified for appraisal and mapped in order to clearly display the activities and objectives included in each phase of the curriculum. Mapping was carried out by two medical students, with access to two academic leads for advice and discussion about decision making where necessary.

### Criteria for viability

In order to be viable for assessment, course components had to achieve the set criteria of being planned, explicit in their content, universal to all students and quantifiable in time, objective or value (Table 2). Whilst some components such as clinical placements contained variable experiences, only the constant elements of these were considered for assessment, for example learning objectives or planned activities for each individual placement.

**Table 2:** Set rationale used in identifying appropriate course content for review

Criterion	Description
Planned	There must be a clear goal and method of execution for this activity
Explicit	Objectives, outcomes or processes must be clearly defined
Universal	Component must apply to all students of relevant year group(s)
Quantifiable	Must be clear in time allocated, outcomes expected or value of the exercise

### Unit of measurement

In order to standardise and quantify the curriculum analysis, the Resilience Outcome (RO) classification system was devised. Learning objectives or activities considered to support the development of resilience through one or more of the 31 HCC factors were assigned one RO to each HCC factor fulfilled.

### Classification

Two specific systems of classification were employed to the entire curriculum based upon activity type. All assessed course content was categorised as either discrete learning objectives (e.g. explicit learning points of a lecture) or components of structured activities (e.g. the elements involved in completing a research module). Content was then appraised according to the following standards;

1. Discrete learning objectives
  Learning objectives identified as influencing resilience were taken from student module guides, phase handbooks and tutor guides. Each identified learning objective was appraised for ROs using the HCC. As numerous learning objectives fulfilled multiple categories, each learning objective was allowed a maximum allocation of 3 ROs. Examples of qualifying learning objectives are shown in Table 3.
2. Components of structured activities
  Structured activities were appraised on the basis of explicitly stated objectives, processes and requirements. These were identified through manual review of handbooks and individual assignment specifications. No RO limit was applied to structured activities due to a notable increase in complexity when compared to discrete learning objectives.

**Table 3:** Examples of learning objectives identified during the HYMS resilience review for years 1–5

Year of study	Identified in each year, course component or learning objective	Criteria mapped	Care criteria descriptor
1	Practice *listening* to a patient’s views and experience	1.1	Personality/temperament/optimism/openness
		4.2	Communication
2	Consult with a simulated patient who has cancer	1.2	Empathy
		6.2	Managing emotions
3	Describe the ethical and practical aspects of recruitment to clinical trials	2.1	Reflective
		3.4	Developing ethical compass
4	Demonstrate the ability to work effectively with other health care professionals	2.2	Self-awareness/insightful
		4.1	Team working ability
5	Manage intravenous patient-controlled analgesia and epidural analgesia and their side effects	3.3	Exec function/organisational abilities
		3.5	Confidence/autonomy
		9.4	Planning abilities

### RO logging

RO counts for all 31 resilience factors of the HCC were manually logged using Microsoft Excel for each learning block across the three phases sequentially. This was executed using a 2-stage rating system. Initial ROs were assigned by a single rater. These were then reviewed and verified by a second rater, with discrepancies being discussed with the project lead. Once all identified course elements had been considered and classified, the completed RO totals were collapsed into the ten parent categories to facilitate comparison and analysis between phases.

## Results

**Fig. 1:**
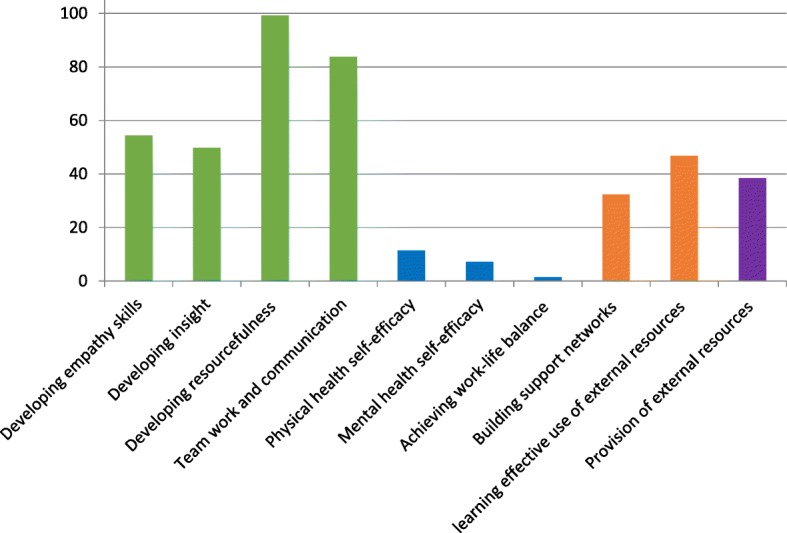
Average number of ROs assigned across the 9 categories over the 5-year MBBS course. The Y-axis values represent the number of ROs assigned per category

RO assignments were logged for each of the 31 resilience factors of the HCC and combined under each of the ten parent categories (e.g. “Developing empathy skills”) to produce quantitative representations of the number of ROs assigned to each. Upon completion of the curriculum review, a total of 2124 ROs were identified (Table 4). These counts were then used to generate an average RO number for each of the ten parent categories over the five years of study (Fig. 1).

**Table 4:** Number of Resilience outcomes identified in each of the ten main categories by undergraduate year

Total number of resilience outcomes (RO) logged, distributed by year of study and CARE category							
RO category	RO number by year of study					Total RO number	Average RO Number per year
	Y1	Y2	Y3	Y4	Y5		
Developing empathy skills	44	40	85	74	29	272	54
Developing insight	36	34	55	50	74	249	50
Developing resourcefulness	86	94	136	129	51	496	99
Team work and communication	94	102	103	82	38	419	84
Physical health self-efficacy	25	5	12	14	1	57	11
Mental health self-efficacy	5	16	10	3	2	36	7
Achieving work-life balance	2	0	0	5	0	7	1
Building support networks	28	24	47	36	27	162	32
learning effective use of external resources	51	47	55	54	27	234	47
Provision of external resources	43	33	61	37	18	192	38

The totals showed a consistent pattern across all three phases of the MBBS course. The category with the highest average LO assignment was “Internal factors”, comprised of subcategories “Developing empathy skills”, “Developing insight”, “Developing resourcefulness” and “Team work and communication”. These subcategories all represent the development of core skills and traits that are vital for successful clinical practice.

The category with the lowest average LO assignment was “Lifestyle factors”, comprised of subcategories “Physical health self-efficacy” (e.g. Nutrition/sleep/physical activity), “Mental health self-efficacy” (e.g. Managing emotions) and “Achieving work-life balance” (e.g. pursuit of hobbies). These subcategories lack the dual functionality of providing both resilience and academic achievement seen in the “Internal factors” section. There were limited learning activities in the curriculum related to these factors.

## Discussion

### Assessing the curriculum

We found that this methodology for assessing the curriculum had a number of advantages. Firstly it allowed for systematic exploration of the curriculum across the full five years exploring resilience from a range of perspectives. It was easy to use and received good feedback from the curriculum planners. The methodology was clear and all participants agreed it would be easy to replicate. Weaknesses included the fact that resilience is a broad concept and therefore different tools or people may interpret elements in the curriculum as being related or unrelated depending on their own views. We sought to address this by using terms in plain English that had face validity such as ‘reflective’ and ‘team working ability’. It also requires time to carefully examine all aspects of the 5 year curriculum. Some medical schools may also have ‘hidden’ curricula activities that would not be visible to assess.

### Responding to the data

The curriculum analysis provided an overview of the 5-year course that could be used to identify and bolster areas of the curriculum that were less well represented in the data. This was carried out through collaboration with numerous senior staff members from the medical school, alongside senior clinicians from the main local NHS trust. This multidisciplinary collaboration resulted in a comprehensive list of short, medium and long-term recommendations, including both modification of existing course elements and the creation of bespoke learning experiences to fulfil the specific needs identified. These recommendations were presented for each of the three course phases, and an additional category comprising of *medical school culture, student support and wellbeing*.

#### Example recommendations; phase 1

- Enhance the Phase I to Phase II transition program in order to buffer the effects of changing to a more placement-based environment and help students develop skills in managing workplace transitions.
- Increase focus on the emotional aspects of medicine through increased essay options. Enhanced reflective essay writing based on placement experiences or interviews with senior healthcare staff including the topic of resilience, self-efficacy and work life balance:
- Opportunities for Balint Groups.

#### Example recommendations; phase 2

- Re-map or expand reflective assignments to enhance the focus on resilience and resilience theory. This could include examples such as structured essays based on self-compassion and self-reflection.
- Extend reflective exercises to incorporate the reality of the healthcare environment and culture. This would include general culture, pressures, staffing levels and their effects, hierarchies and attitudes of more senior medics. This could take the form of structured or unstructured essays, reflective diary keeping or group discussion-based environments. Problem solving, assertiveness, whistleblowing skills and processes could be incorporated with opportunities for Schwartz rounds to promote open discussion skills.

#### Example recommendation; phase 3

Negative mental health effects surrounding making mistakes or facing scrutiny may be more pronounced in high-achieving students such as medics. Current literature could guide the construction of a framework to teach students to mediate the emotional impact of making errors or mistakes, facing scrutiny and receiving complaints (managing emotions).

#### Example recommendations; culture, student support and wellbeing

- Increase focus and communication of the medical school educational philosophy, ethos and values in order to bolster connectedness. This includes ongoing work into engaging with the student voice and developing good lines of communication
- Run workshops teaching emotional wellbeing skills and techniques such as mindfulness and meditation. These could be offered to year groups, placement groups, PBL groups or open signup for students of all years. These workshops could be designed to be incorporated into learning blocks such as psychological health or palliative care

**Fig. 2:**
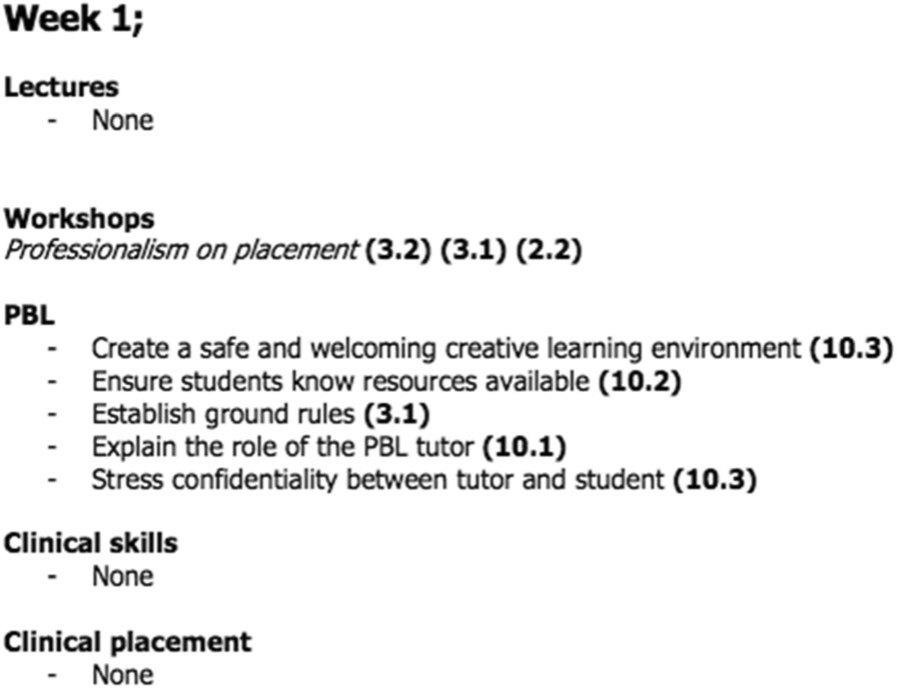
Excerpt from the standalone curriculum for resilience. This section represents year one, week one. The curriculum is organized to display different activity types, and the learning objectives found within

Second to providing the basis for discussion, analysis and action planning, the data sets were collated into an itemised, standalone curriculum detailing week-by-week activities and experiences that possess elements of resilience building (see Fig. 2 for an example). This was undertaken for the entire five-year course, resulting in a complete directory of resilience outcomes (learning outcomes with a clear resilience component). It is hoped that this separate curriculum will prove a useful tool in both cataloguing and further enhancing the undergraduate course by providing a complete record of resilience building activities, and additionally maintaining focus on this aspect of medical education through enhanced visibility and ease of access.

## Conclusions

We found that this methodology was a straightforward way of assessing a medical school curriculum. It came with a helpful blend of theoretical underpinnings and down to earth applicability and this appeared to enhance engagement with curriculum developers and teachers.

We found the concept of resilience to be very broad in the general literature. We have tried to be clear about those experiences that promote coping, adaptability, resourcefulness and enhanced empathy and self-compassion skills, using the acronym CARE to encapsulate this. By contrast the medical literature can be very narrow in what it considers intervention to promote resilience, with one recent systematic review’s main findings focusing on psychosocial skills training and mindfulness [33]. We would encourage educators to think broadly, incorporating and building upon the factors detailed in the HCC (Table 1), and for there to be further research to refine this objective.

The detailed evaluation of the curriculum was helpful in allowing us to visualise the strengths and opportunities for improvement in terms of teaching resilience and resourcefulness. The results showed that the curriculum could be improved in a number of areas, especially emotional wellbeing and physical health support, and in particular supporting students to develop a healthy work life balance. Various medical schools are seeking to address this issue. On many occasions this is using voluntary additional elements to the curriculum [28]. We would argue that this should be directed at all medical students and not optional, and medical students should be actively involved in planning.

Importantly we have found that this piece of work has prompted discussion across the medical school that has been profoundly productive, and wide ranging. This includes the responsibility of the medical school to provide the necessary processes and to support students to carefully consider roles of the future and the curriculum in promoting resilience. Studies suggest that doctors experiencing burnout are more likely to use ineffective coping strategies [34]. It would be beneficial, therefore to infuse medics with more positive, varied and comprehensive coping tools during their education. It is hoped that both the provision and normalisation of these tools and activities paves the way for more competent, rational and progressive coping strategies in both students and practicing medics.

## Data Availability

Although we have provided summary data, the full data sets collected during this research is available upon request of the authors.

## Abbreviations

HCC: HYMS Care criteria
RO: Resilience outcome
